# Overexpression of *mqsR* in *Xylella fastidiosa* Leads to a Priming Effect of Cells to Copper Stress Tolerance

**DOI:** 10.3389/fmicb.2021.712564

**Published:** 2021-09-20

**Authors:** Isis Gabriela Barbosa Carvalho, Marcus Vinicius Merfa, Natália Sousa Teixeira-Silva, Paula Maria Moreira Martins, Marco Aurélio Takita, Alessandra Alves de Souza

**Affiliations:** ^1^Centro de Citricultura Sylvio Moreira, Instituto Agronômico, Cordeirópolis, Brazil; ^2^Department of Entomology and Plant Pathology, Auburn University, Auburn, AL, United States

**Keywords:** persister cells, toxin-antitoxin (TA), phytopathogenic bacteria, copper tolerance system, stress adaptation

## Abstract

Copper-based compounds are widely used in agriculture as a chemical strategy to limit the spread of multiple plant diseases; however, the continuous use of this heavy metal has caused environmental damage as well as the development of copper-resistant strains. Thus, it is important to understand how the bacterial phytopathogens evolve to manage with this metal in the field. The MqsRA Toxin–Antitoxin system has been recently described for its function in biofilm formation and copper tolerance in *Xylella fastidiosa*, a plant-pathogen bacterium responsible for economic damage in several crops worldwide. Here we identified differentially regulated genes by *X. fastidiosa* MqsRA by assessing changes in global gene expression with and without copper. Results show that *mqsR* overexpression led to changes in the pattern of cell aggregation, culminating in a global phenotypic heterogeneity, indicative of persister cell formation. This phenotype was also observed in wild-type cells but only in the presence of copper. This suggests that MqsR regulates genes that alter cell behavior in order to prime them to respond to copper stress, which is supported by RNA-Seq analysis. To increase cellular tolerance, proteolysis and efflux pumps and regulator related to multidrug resistance are induced in the presence of copper, in an MqsR-independent response. In this study we show a network of genes modulated by MqsR that is associated with induction of persistence in *X. fastidiosa*. Persistence in plant-pathogenic bacteria is an important genetic tolerance mechanism still neglected for management of phytopathogens in agriculture, for which this work expands the current knowledge and opens new perspectives for studies aiming for a more efficient control in the field.

## Introduction

*Xylella fastidiosa* is a phytopathogen with a broad host range that affects plants worldwide (Almeida et al., 2019). Plant diseases caused by this bacterium include citrus variegated chlorosis (CVC), Pierce’s disease (PD) in grapevines, and the olive quick decline syndrome (OQDS), which constitute important threats for these crops (Almeida et al., 2019; Saponari et al., 2019; Coletta-Filho et al., 2020). Copper-based compounds are widely used in agriculture as a chemical strategy to limit the spread of multiple plant diseases (Lamichhane et al., 2018). Although *X. fastidiosa* is not itself controlled by copper spraying, biocomplexes containing copper, zinc, and citric acid have been used to control *X. fastidiosa* in olive groves (Girelli et al., 2019). Copper has an important contribution in crop protection; however, there are many issues related to the use of this heavy metal such as phytotoxicity, soil accumulation, negative effects on soil biota, and development of copper-resistant strains (Lamichhane et al., 2018). Thus, regarding plant-pathogen interaction, it is important to understand how the bacterial phytopathogens evolve to deal with this metal in the field.

In *X. fastidiosa*, the *mqsRA* toxin–antitoxin (TA) system type II is a genetic mechanism that has been associated with tolerance to copper stress (Muranaka et al., 2012). There are six types of TA system, which are distinct according to the action, nature, and mechanisms used by the antitoxins to neutralize the activities of the toxins (Page and Peti, 2016). Typically, in these systems, the toxin gene product is a protein and the antitoxin gene is a non-coding RNA (in types I and III) or a protein (in types II, IV, V, and VI) (Page and Peti, 2016; Harms et al., 2018). Bacterial toxin-antitoxin (TA) systems encode a stable toxin that disrupts cellular function and its labile cognate antitoxin in the same operon. The antitoxin neutralizes toxin activity under normal conditions, while proteases degrade the antitoxin under stress, allowing the toxin activity (Wang and Wood, 2011; Fisher et al., 2017). Moreover, the antitoxin usually regulates the expression of its own TA operon by binding to a palindromic sequence in the promoter region and repressing its transcription (Wang et al., 2011). TA systems have been shown to play a role in persistence, biofilm formation, cell movement, pathogenicity, DNA maintenance, and phage-defense (Wang et al., 2011; Wen et al., 2014; Shidore and Triplett, 2017). In addition, they are highly expressed in persister cells and, thus, are generally responsible for the persistence phenotype (Wang and Wood, 2011; Fisher et al., 2017). A persister cell constitutes a tolerant cell (Lewis, 2010) originating from a population that displays antibiotic persistence, being a subpopulation phenomenon (sometimes referred to as heterotolerance) (Balaban et al., 2019), while, a tolerant cell is the capacity of an entire population of bacteria to survive a bactericidal antibiotic exposure (Balaban et al., 2019). Multidrug resistance in bacteria can occur by distinct ways like the accumulation of the resistance factors like plasmids or genes, each one encoding for resistance to a particular agent, and can or cannot occur along with the activity of multidrug efflux pumps (Nikaido, 2009).

The *mqsRA* TA system was originally described in *Escherichia coli* and shown to be involved in biofilm and persister cell formation (Wang and Wood, 2011). The toxin *mqsR* was the most induced gene in *E. coli* persisters and the first TA system to reduce persister formation upon deletion, while increasing this phenotype after overexpression (Kim and Wood, 2010). It has been demonstrated that *X. fastidiosa* may form persister cells under copper stress (Muranaka et al., 2012; Merfa et al., 2016), representing an important survival strategy still unexplored in plant pathogenic bacteria (Martins et al., 2018).

The MqsRA TA system is composed of the MqsR toxin, which is an endoribonuclease that degrades messenger RNA (mRNA) with GCU motifs and the MqsA antitoxin that binds and inactivates the toxin via its N-terminal domain (Brown et al., 2009; Yamaguchi et al., 2009; Lee et al., 2014). Due to its ability to selectively degrade mRNA, MqsR also acts as a global regulator (Wood et al., 2013). Thus, aiming to identify genes modulated by MqsR in *X. fastidiosa*, we overexpressed this toxin under the control of its native promoter and performed RNA-Seq when growing cells under normal and upon copper stress conditions.

Our results show that MqsR is a key gene regulator in the pathway tolerance of *X. fastidiosa* to copper stress, mediating several genes that prompt the cells to enter in a state that suggests the formation of persisters. In addition, copper induces MqsR-independent responses related to proteolysis and multidrug resistance through transcriptional regulator, transporters, and efflux pumps in order to increase the bacterial tolerance to this metal. This study presents unexplored mechanisms in phytopathogens that could have important impacts on how they can deal with agrochemicals and highlight the persistence phenomenon that could be occurring in the field.

## Materials and Methods

### Bacterial Strains and Transformation

The bacterial strains used in this study were the *X. fastidiosa* wild-type strain 11399 (Coletta-Filho et al., 2001; Niza et al., 2016) and 11399 overexpressing *mqsR* under the control of its native promoter (*Xf-mqsR*) (Merfa et al., 2016) (Supplementary Table 1). The increased amount of MqsR was previously confirmed by Western blot (Merfa et al., 2016). We transformed *X. fastidiosa* 11399 strain with the pXF20 empty vector (Lee et al., 2010), by electroporation (1.8 kV, 200 Ω, 25 μF) to serve as negative control (*Xf*-EV). The transformants were grown on selective medium PWG (phytone peptone; BD Biosciences, San Jose, CA, United States) 4.0 (g/L), trypticase peptone (BD) 1.0 (g/L), K_2_HPO_4_ (Sigma, St. Luis, MO, United States) 1.2 (g/L), hemin chloride stock (Sigma) 10 (mL/L), KH_2_PO_4_ 1.0 (g/L), Gelzan (Sigma) 8.0 (g/L), MgSO_4_:7H_2_O, 0.4 (g/L), phenol red stock (Sigma; 0.2% (w/v) phenol red in distilled water) 10 (mL/L), glutamine (Sigma) 4 (g/L), and bovine serum albumin fraction-five (BSA) (Sigma) 3 (g/L); this medium was prepared according Davis et al. (1981) plates supplemented with 50 μg/mL kanamycin. The transformation was confirmed by PCR using a specific pair of primers to detect the pXF20 plasmid (Supplementary Figure 1). The primers used to confirm this transformation are oriV-pXF20-F 5’-GGTTTGTGAAAGCGCAGTG and trfA-pXF20-R 5’-ATTGCCAATTTGGACAGATG. The *Xf*-EV and *Xf-mqsR* strains were routinely grown on selective PWG plates supplemented with 50 μg/mL kanamycin at 28°C for 7 days.

### Copper Sensitivity Assay

To evaluate the effects of copper on *X. fastidiosa* growth and formation of persisters, *Xf*-EV and *Xf-mqsR* cells were grown in PW broth (PWG without Gelzan) (Davis et al., 1981) and treated with 3 mM CuSO_4_.5H_2_O (Sigma). Control samples of both strains were grown in non-copper PW broth. Cells grown on solid PW were harvested from plates, resuspended in PBS buffer, and the optical density (OD_600 *nm*_) was adjusted to 0.3 and inoculated into PW broth to grow for another 7 days. The cells were then collected and the OD_600 *nm*_ was adjusted to 0.1. From each of these bacterial suspensions, 10-mL aliquots were inoculated into 90 mL fresh PW broth and incubated at 28°C for 14 days at 150 rpm. Subsequently, *Xf*-EV and *Xf-mqsR* cells were exposed to 0 (“C-0” for *Xf*-EV, and “M-0” for *Xf-mqsR*) and 3 mM copper (“C-3” for *Xf*-EV and “M-3” for *Xf-mqsR*) for 24 h (Merfa et al., 2016). Each treatment was performed in duplicates for each strain. The cells of each culture were collected, rinsed with DEPC water, and resuspended in 11 mL of PBS buffer. An aliquot of 1 mL from each suspension was used to determine colony formation units (CFU/mL), and to perform electron microscopy analysis, as described below. The cells of the remaining 10 mL were collected under the same conditions and stored at −80°C for RNA extraction. Three independent biological replicates were performed.

### Bacterial Growth Under Copper Stress

Aliquots of the entire experimental condition described above (Supplementary Figure 2) were collected to determine the CFU/mL of each biological experiment at the following time course: inoculation time (t0), 14 days after growth (stationary phase, Campanharo et al., 2003) in fresh PW broth when copper was added (t1) and 24 h after copper treatment (t2), completing 15 days of growth. From each sample, a 10-fold serial dilution was performed and plated in PWG to estimate CFU. Four replicates were used for each sample, which were grown at 28°C for 30 days. The measurements were performed in triplicates, and results were scored as the means ± standard deviation and compared using the Student’s *t*-test (*p* ≤ 0.05).

### Scanning Electron Microscopy

Scanning electron microscopy was performed under the experimental conditions described above (Supplementary Figure 2). Briefly, an aliquot of the planktonic and biofilm cells was sampled 24 h after copper addition for each *X. fastidiosa* strain. Controls without copper were also collected for both *Xf*-EV and *Xf-mqsR*. Samples were centrifuged and resuspended in RNA Later solution (Thermo Fisher Scientific, Waltham, MA, United States), frozen in liquid nitrogen and stored at −80°C. For microscopy analysis, cells were thawed, centrifuged, fixed in 2.5% glutaraldehyde in 0.2 M sodium cacodylate buffer (v/v) and kept at 4°C until use. Preparation of samples for visualization was done according to Kozlowska et al. (2014). Electron micrographs were captured with a magnification of 4000 × using a Hitachi TM 3000 scanning electron microscope (Hitachi, Tokyo, Japan). The *Xf*-EV and *Xf-mqsR* cells were measured using the ImageJ software (ImageJ, 2018) to determine the length and proportion of elongated and small cells at 100 cells per treatment. Only cells longer than 4.0 μm were considered elongated (Liu et al., 2014; Merfa et al., 2016), while only cells with a length smaller than 2.0 μm were considered small. The length of each cell in each treatment was analyzed through comparison of means by one-way analysis of variance (ANOVA) followed by Holm–Sidak multiple comparison test or Tukey’s HSD test (*p* ≤ 0.05).

### RNA Isolation and RNA-Seq

RNA-Seq reads were produced from 12 RNA samples: three from non-treated *Xf*-EV cells, three from non-treated *Xf-mqsR*, three from copper-treated cells of *Xf*-EV, and three from copper-treated *Xf-mqsR* cells. Total RNA was extracted using the hot phenol method (Khodursky et al., 2003), treated with DNase I RNase free (Qiagen, Hilden, Germany), purified using the RNeasy Plus Kit (Qiagen, Hilden, Germany) and eluted in 30 μL of RNase-free water. Concentrations were determined by spectrophotometry (NanoDrop 8000, Thermo Fisher Scientific). Ribo-Zero rRNA^TM^ Removal Kit (Illumina, San Diego, CA, United States) was used for rRNA removal. The depleted RNA was precipitated using ethanol according to the manufacturer’s instructions and resuspended in 10 μL of RNase-free water. Samples were quantified for the presence of rRNA using the 2100 Bioanalyzer system (Agilent Technologies, Santa Clara, CA, United States) at the Life Sciences Core Facility (LaCTAD). cDNA libraries were prepared using the Illumina TruSeq Stranded mRNA Library Prep Kit (Illumina). Sequencing was performed using the HiSeq High Output kit (Illumina) on a HiSeq 2500 system (Illumina), run with 2 × 100 bp paired-end reads.

### RNA-Seq Data Analysis

The sequencing reads were analyzed in the FastQC program (Wingett and Andrews, 2018) and processed using Trimmomatic (Bolger et al., 2014) to remove adapters and extremities with poor quality. The reads were mapped to the genome of *X. fastidiosa* 9a5c (NCBI BioProject accession PRJNA271) using the STAR program (Dobin et al., 2013). From the mapped data, the gene-mapped reads were counted using the Subread package (Liao et al., 2019). Standardization and analysis of differential gene expression (*p* < 0.05) was performed using the EdgeR package (Robinson et al., 2010), computed using data from all three biological replicates. Differentially expressed genes obtained from EdgeR analyses were used for functional categorization by Blast2GO (Götz et al., 2008). Venny 2.1.0 (Oliveros, 2007) was used to show exclusive genes regulated in *Xf-mqsR* under copper stress.

### Data Validation by Quantitative Real Time-PCR (RT-qPCR)

RNA samples were obtained from three other experiments using the same experimental condition as the RNA-Seq. A total of 250 ng of purified RNA from each condition was used as input for cDNA synthesis with the Reverse Transcription System kit (Promega, Madison, WI, United States). RT-qPCR was performed using the GoTaq qPCR Master Mix (Promega) in an ABI PRISM 7500 Sequence Detection System (Applied Biosystems, Foster City, CA, United States). Relative expression values were normalized to the *X. fastidiosa* 16S ribosomal RNA endogenous control (Merfa et al., 2016). Cycling parameters were performed according to the manufacturer’s protocol. The relative expression quantification (RQ) was calculated as previously described (Livak and Schmittgen, 2001). The selected genes are based on the RNA-Seq analysis of M-0 and M-3 (Supplementary Table 2). Three independent biological replicates were used for data validation.

### Palindrome Search

The motif 5’-ACC (N)7 GTT-3’ (Merfa et al., 2016), used as target sequence for DNA binding by the antitoxin MqsA, was searched in the genome of *X. fastidiosa* strain 9a5c using PATLOC (Mr̀azek and Xie, 2006), and also at the differentially expressed genes (DEG) data set herein generated.

## Results

### MqsR Overexpression Changes *X. fastidiosa* Phenotype

To evaluate the effects of copper on wild-type *X. fastidiosa* and the *mqsR*-overexpressing strain, bacterial growth with and without copper was evaluated. In a previous work we verified that overexpression of MqsR increased the formation of persister cells under 3 mM of copper stress (Merfa et al., 2016). Here, to access the phenotypic and genetic regulation mediated by MqsR, we used the same condition, where copper was added after 15 days of bacterial growth.

**FIGURE 1:**
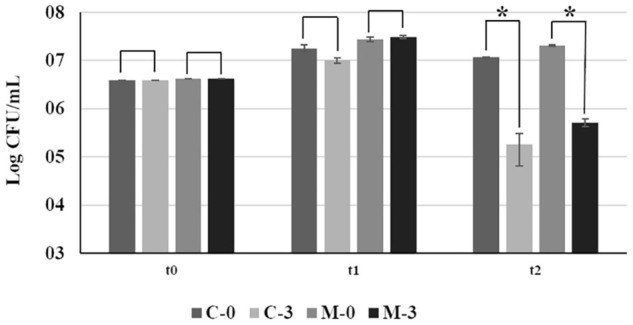
Bacterial growth of *Xf*-EV and *Xf-mqsR* under normal conditions and after copper stress at different time points. C-0: *Xf*-EV; C-3: *Xf*-EV + 3 mM CuSO_4_; M-0: *Xf-mqsR*; M-3: *Xf-mqsR* + 3 mM CuSO_4_. t0: initial inoculum; t1: 14 days of growth; t2: 24 h after copper treatment, at the end of 15 days. Three independent experiments were performed with similar results. * indicates statistical significance by Student’s *t*-test (*p* ≤ 0.05, *n* = 4).

At the time of the inoculation (t0), and after 15 days of growth in fresh PW broth (t1), no significant difference in bacterial growth was observed between C-0 and M-0 (Figure 1). However, 24 h after addition of copper (t2), there was a significant reduction in population size of approximately 100-fold between copper-treated samples (C-3 and M-3) and their respective untreated controls (C-0 and M-0) (Figure 1). We observed an approximately 10% increase in cell survival after copper treatment in populations overexpressing *mqsR* (M-3) in comparison to the control (C-3). However, the difference in CFU counts between M-3 and C-3 was not significant (F = 0.06, *p* = 0.11) (Figure 1).

**FIGURE 2:**
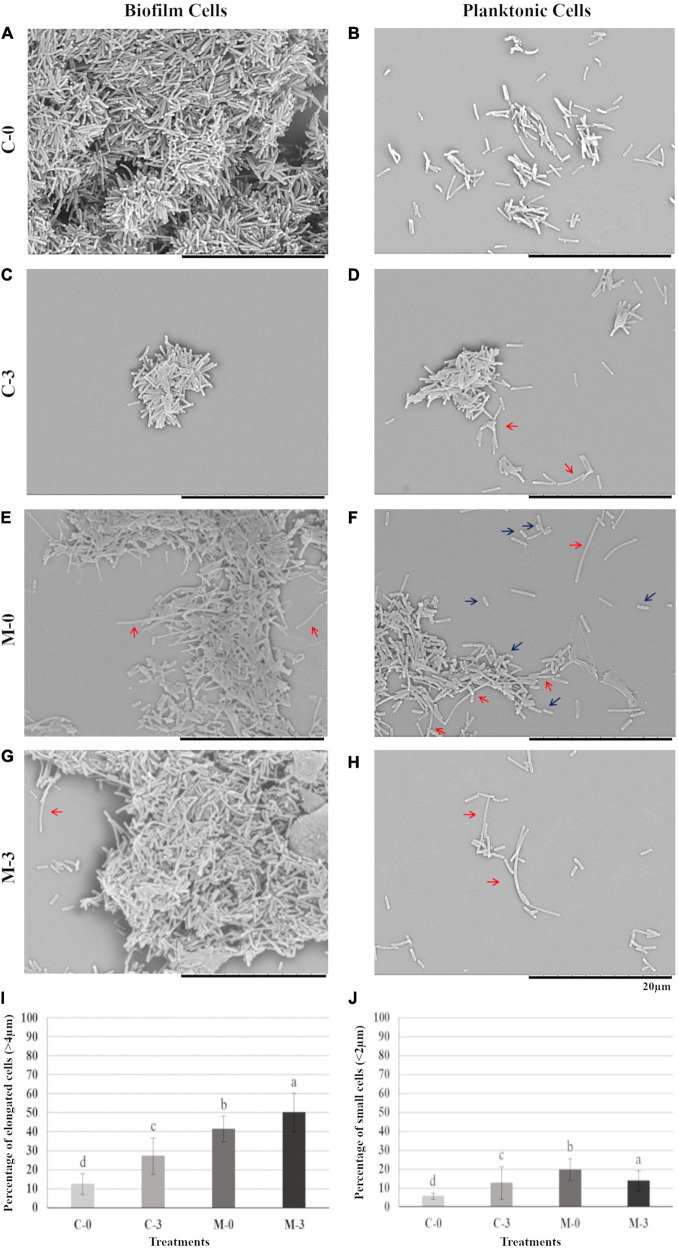
Biofilm and planktonic behavior of *Xf*-EV and *Xf-mqsR* cells under copper stress. Left column: cells in biofilm. Right column: planktonic cells. **(A,B)** Representative pictures of C-0: *Xf*-EV cells without copper treatment. **(C,D)** Representative pictures of C-3: *Xf*-EV cells treated with 3 mM CuSO_4_. **(E,F)** Representative pictures of M-0: *Xf-mqsR* cells without copper treatment. **(G,H)** Representative pictures of M-3: *Xf-mqsR* cells treated with 3 mM CuSO_4_. Red arrows show elongated cells and blue arrows show short cells. Scale bar: 20 μm. **(I)** Percentage of planktonic cells longer than 4.0 μm. **(J)** Percentage of planktonic cells shorter than 2.0 μm in the different treatments. Different letters on top of column bars indicate significant difference as analyzed by one-way ANOVA in SigmaPlot followed by Tukey’s HSD test (*p* ≤ 0.05; *n* = 3 biological replicates, with 100 internal replicates each). C-0: *Xf*-EV cells without copper treatment; C-3: *Xf*-EV cells treated with 3 mM of CuSO_4_; M-0: *Xf-mqsR* cells without copper treatment; M-3: *Xf-mqsR* cells treated with 3 mM of CuSO_4_.

To verify possible phenotypic changes in *X. fastidiosa* cells potentially caused by the overexpression of *mqsR* and copper treatment, samples from each experimental condition were used for scanning electron microscopy. Under normal growth condition, biofilm and planktonic cells in C-0 did not show any significant morphological change (Figures 2A,B). However, when copper was added (C-3) a reduction was observed in biofilm size (Figure 2C), and curiously, copper induced aggregation and elongated cells in the planktonic condition (Figure 2D, red arrows).

On the other hand, *X. fastidiosa* overexpressing *mqsR* (M-0) presented more elongated cells even without copper stress (Figures 2E,F, red arrows), and at an even greater extent than C-3 (Figure 2D, red arrows). In addition, *X. fastidiosa* overexpressing *mqsR* (M-0) displayed a phenotypic heterogeneity that can be demonstrated by the presence of a higher population of shorter cells when compared to the other treatments (Figure 2F, blue arrows). Elongated cells were also observed in *X. fastidiosa* overexpressing *mqsR* in presence of copper (M-3) in both biofilm and planktonic conditions (Figures 2G,H, red arrows).

Microscopy images for each condition (*n* = 100) were used for counting elongated and short cells in the planktonic fraction (Figures 2I,J). The results showed a good agreement with the visual observation, with a higher population of elongated cells in *X. fastidiosa* overexpressing *mqsR* (M-0) compared to C-0. Copper induced an increase of elongated cells in both populations (C-3 and M-3). Interestingly the number of elongated cells in M-0 is naturally even higher than C-3 (Figure 2I). Similarly, higher percentages of short cells were observed in presence of copper (C-3 and M-3) or *X. fastidiosa* overexpressing *mqsR* (M-0) (Figure 2J).

Overall, these results show that besides copper treatment, *mqsR* overexpression also led to changes in *X. fastidiosa* morphology and pattern of aggregation, culminating in a global phenotypic heterogeneity. Interestingly, heterogeneous phenotypes in single bacterial populations have been described as indicative of persister cells (Michiels et al., 2016; Fisher et al., 2017).

### RNA-Seq Data

RNA-Seq reads were produced for C-0, C-3, M-0, and M-3 (Supplementary Figure 3). Raw sequencing reads were deposited under the NCBI Bio-Project ID PRJNA718853. Average post-trim read length ranged from 36 to 105 bp, the reads aligned to the genome of *X. fastidiosa* 9a5c. Variable rRNA and small RNA depletion efficiencies between samples resulted in 0–7.3% in library preparation. Mapped reads were used to determine transcript boundaries and normalized expression for all protein-coding genes by EdgeR (Supplementary Material 1, Data Sets 1–5). Pearson’s correlation coefficient for protein-coding gene expression between experimental replicates ranged from 0.89 to 0.93. Highlighted DEGs of libraries were characterized according to the biological process by Blast2GO (Supplementary Material 1, Data Sets 6–11).

### *mqsR* Differentially Modulates Global Gene Expression of *X. fastidiosa*

To investigate global expression changes likely to be associated with the phenotypes described above, we performed RNA sequencing analysis. To identify which genes were modulated by MqsR under normal growth conditions, we assessed the pairwise comparison between M-0/C-0 libraries (without copper treatment). Amongst the DEGs, 189 genes showed upregulation by the overexpression of *mqsR* alone, while 164 genes were downregulated (Supplementary Material 1, Data Set 1; *p* < 0.05). RNA-Seq expression values (log_2_ fold-change) were confirmed by RT-qPCR for 10 selected genes based on Table 1, with a Pearson correlation coefficient of 0.89 (Supplementary Figure 4). According to the data obtained through RNA-Seq (Supplementary Material 1, Data Set 1), the selected genes that are possibly modulated by MqsR are listed in Table 1.

**TABLE 1:** Genes modulated by MqsR in *X. fastidiosa.*

**Functional group**	**Gene name** *	**Locus Tag** **	**Protein**	**Product**	**LogFC**
**Peptide metabolic process**	Chaperone protein *clpB*	XF_RS01600	WP_010892912.1	Chaperone protein ClpB	–1.47
	Molecular chaperone	XF_RS00340	WP_010892630.1	Molecular chaperone	1.66
**Proteolysis**	ATP-dependent Clp protease proteolytic subunit	XF_RS05040	WP_010893698.1	ATP-dependent Clp protease proteolytic subunit	–1.08
	ATP-dependent Clp protease ATP-binding subunit *clpA*	XF_RS06080	WP_010893944.1	ATP-dependent Clp protease ATP-binding subunit ClpA	–0.878
	Peptidase S14	XF_RS02140	WP_042462775.1	Clp protease ClpP	–0.70
	Protease HtpX	XF_RS11410	WP_010895042.1	Protease HtpX	–1.30
**Cell division**	Hypothetical protein *(rlpA)*	XF_RS09450	WP_010894633.1	Septal ring lytic Transglycosylase RlpA family protein	–1.11
**Toxins**	Hypothetical protein	XF_RS01135	WP_010892803.1	Hypothetical protein	4.33
	(Colicin V)				
	Bacteriocin	XF_RS10410	WP_010894853.1	Bacteriocin	0.680
**Regulatory functions**	Transcriptional regulator	XF_RS07310	WP_010894181.1	Transcriptional regulator	2.25
	LysR family transcriptional regulator	XF_RS07605	WP_031336630.1	LysR family transcriptional regulator	2.74
	DNA-binding response regulator	XF_RS01630	WP_004083627.1	DNA-binding response regulator ompR	–0.843
	AraC family transcriptional regulator	XF_RS05305	WP_010893760.1	AraC family transcriptional regulator	–0.864
	Hypothetical protein	XF_RS07050	WP_042463203.1	Hypothetical protein (Helix-turn-helix XRE-family like proteins)	2.25
	RNA polymerase-binding protein *dksA*	XF_RS04240	WP_010893509.1	RNA polymerase-binding protein DksA	–0.85
	DNA-directed RNA polymerase subunit alpha	XF_RS04985	WP_004090142.1	DNA-directed RNA polymerase subunit alpha	1.32
	(*rpoA*)				
	DNA-directed RNA polymerase subunit omega (*rpoZ*)	XF_RS06345	WP_010894003.1	DNA-directed RNA polymerase subunit omega	1.10
	RNA-binding protein Hfq	XF_RS00365	WP_010892636.1	RNA-binding protein Hfq	1.11
**Attachment/motility**					
**Fimbrial adhesins**	Fimbrial protein	XF_RS00335	WP_010892629.1	Fimbrial biogenesis outer membrane usher protein	1.21
	(*fimD*)				
**Transporters**	Membrane protein *(tolC)*	XF_RS11265	WP_010895004.1	Membrane protein	0.755
	multidrug transporter	XF_RS09045	WP_010894536.1	AcrB/AcrD/AcrF family protein	0.809
**TA system**	Addiction module antidote protein	XF_RS12375	WP_010895238.1	DNA-binding protein	–1.94
	Plasmid stabilization protein *(parE)*	XF_RS09000	WP_010894527.1	Type II toxin-antitoxin system RelE/ParE family toxin	1.13
	Antitoxin (*mqsA*)	XF_RS10795	WP_010894926.1	Antitoxin	1.06
	HP (*mqsR*)	XF_RS10790	WP_010894925.1	Type II toxin-antitoxin system MqsR family toxin	4.47
	Addiction module protein	XF_RS12370	WP_004091397.1	Type II toxin-antitoxin system RelE/ParE family toxin	–2.41
	Cytotoxic translational repressor of toxin-antitoxin stability system (*relE*)	XF_RS12805	WP_080507186.1	RelE_type II toxin-antitoxin system RelE/ParE family toxin	–0.823
**Quorum sensing**	Long-chain fatty acid–CoA ligase (*rpfB*)	XF_RS01220	WP_010892826	Chemical binding	0.789

*Nomenclature according to GenBank.

**Locus Tag corresponds to GenBank accession numbers.

**FIGURE 3:**
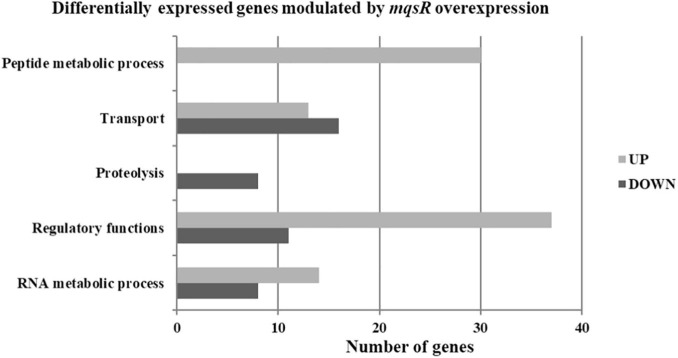
Gene ontology categorization of differentially expressed genes in *X. fastidiosa* in response to overexpression of *mqsR*. Genes were classified by functional category of biological processes using Blast2GO. UP: upregulated genes; DOWN: downregulated genes.

Functional categorization of these 353 DEGs comprised genes associated with peptide metabolic process, transport, proteolysis, transcriptional regulation, and RNA metabolic processes (Figure 3, Supplementary Material 1, and Data Sets 6, 7). Genes associated with proteolysis were exclusively downregulated, including the proteases *clpA* and *clpP*. On the other hand, genes related to peptide metabolism were exclusively upregulated, including those related to ribosomal subunit scaffolding of RNA polymerase (RNAP), such as *rpoA* and *rpoZ*. These genes are also listed in the regulatory function category, together with *mqsR*, *lysR*, and the post-transcriptional regulator *hfq*, which were induced. The regulators genes *mqsR*, *rpoZ*, and *lysR* are related to bacterial survival, stress responses, and pathogenicity (Maddocks and Oyston, 2008; Santiago et al., 2015; Merfa et al., 2016; Weiss et al., 2017). *rpoZ* mutants of *Mycobacterium smegmatis* were deficient in motility and biofilm formation, consequently affecting the formation of extracellular matrix (Mathew and Chatterji, 2006). Besides, the overexpression of transcriptional regulator type LysR from *X. fastidiosa* in *E. coli* was described to a play role in maturation of biofilm during its development (Santiago et al., 2015). In *X. fastidiosa*, it is important to emphasize that the formation of biofilm is characterized as the main pathogenicity mechanism (Coletta-Filho et al., 2020). The transport category included the upregulation of *tolC* and *acrB*, both related to efflux pumps (Weston et al., 2018) and bacterial persistence (Pu et al., 2016). Efflux pumps are important for broad cellular homeostasis during stress responses. They export a wide variety of compounds, such as signaling molecules and antimicrobial compounds (Langevin and Dunlop, 2018). *rlpA*, a gene involved in cell division (Jorgenson et al., 2014; Berezuk et al., 2018), was downregulated, and *fimD*, which is involved with type I fimbrial adhesin (Meng et al., 2005), was upregulated. These genes are involved in bacterial physiology and biofilm formation, respectively. Furthermore, our results showed TA-related genes. The *relE* (XF_RS12805) toxin was downregulated by MqsR; this gene is associated with inhibition translation by cleavage of mRNA in the ribosome (Fiebig et al., 2010). Another toxin, *parE* gene, was upregulated and it is responsible for inhibiting gyrase and thereby blocks chromosome replication (Jiang et al., 2002). Modulation of *relE* and *parE* suggests that these bacterial cells maintain basal activities with reduced metabolism as shown in persister cells (Lewis, 2007). In addition, the repression of *rlpA*, inhibiting cell division and induction of the toxin encoding *parE* (Yuan et al., 2011), which inhibits bacterial division, could contribute with the observed elongated phenotype. Taken together, these observations suggest that overexpression of *mqsR* contributes to bacterial survival during stress response by activating pathogenicity regulators and inhibiting proteolysis and cell division.

### Overexpression of *mqsR* Modulates Translation in *X. fastidiosa* Under Copper Stress

To identify the influence of copper on the gene expression, the following pairwise comparisons of the sequencing libraries were performed: i. M-3/M-0, and ii. C-3/C-0. Each pairwise comparison generated 417 and 662 DEGs, respectively. The M-3/M-0 comparison resulted in 238 upregulated and 179 downregulated genes (Supplementary Material 1, Data Set 2), while the C-3/C-0 analysis resulted in 335 upregulated and 327 downregulated genes (Supplementary Material 1, Data Set 3).

**FIGURE 4:**
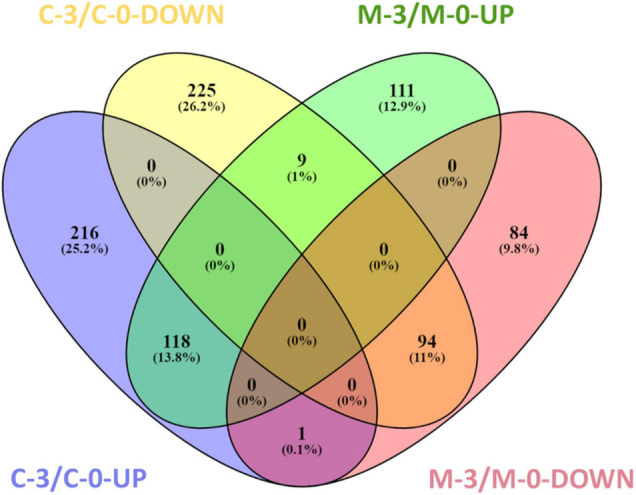
Venn diagram representing the number of up and downregulated genes in *X. fastidiosa* under copper stress. M-3/M-0: pairwise comparison between the M-3 (*Xf-mqsR* + CuSO4) and M-0 (*Xf-mqsR*) libraries. C-3/C-0: pairwise comparison between the C-3 (*Xf*-EV + CuSO4) and C-0 (*Xf*-EV) libraries. UP: upregulated genes; DOWN: downregulated genes.

To verify *X. fastidiosa* genes modulated by MqsR in response to copper stress, a Venn diagram was used to compare the up- and downregulated genes in the M-3/M-0 and C-3/C-0 libraries (Figure 4). This comparison provided genes modulated only by *mqsR*-overexpressing cells under copper stress (M-3/M-0), resulting exclusively in 111 upregulated and 84 downregulated genes (Figure 4 and Supplementary Material 1, Data Set 4).

In the search for genes oppositely modulated between *Xf*-EV (C-3/C-0) and *Xf-mqsR* (M-3/M-0), a set of nine genes were found to be downregulated in C-3/C-0 and upregulated in M-3/M-0. Of those, there is the *yeiP* elongation factor (XF_RS09585), three ribosomal subunits (XF_RS00715, XF_RS00285, XF_RS09575), the *msrB* (XF_RS03590) and *yuxK* (XF_RS04035), the aminotransferase *astC* (XF_RS06015, also known as *argM* or *cstC*), and two hypothetical proteins (XF_RS03860, XF_RS05645). Interestingly though, the ribosomal protein (XF_RS12125) was the only one showing an opposite behavior, being induced in C-3/C-0 but suppressed in M-3/M-0. This protein is involved in translation, and accordingly, this category was downregulated in M-3/M-0. In *E. coli*, it is known that persister cells have very low metabolism, with non-growing cells as a result of a depletion in translation and, thus, in protein production capacity, cessation of transcription and reduction in ATP production (Kwan et al., 2013; Kim et al., 2018). Moreover, the ability to wake up from this persister state was related to ribosome content (Kim et al., 2018). Our results show categories such as peptide metabolic processes and translation downregulated, suggesting low-metabolism and depletion of protein production in *X. fastidiosa* in such condition.

Finally, 94 genes remained downregulated, and 118 genes were upregulated in both conditions. We believe that these sets of genes are modulated due to treatment with copper itself and are likely to be independent of MqsR functions (Supplementary Material 1, Data Set 5).

**FIGURE 5:**
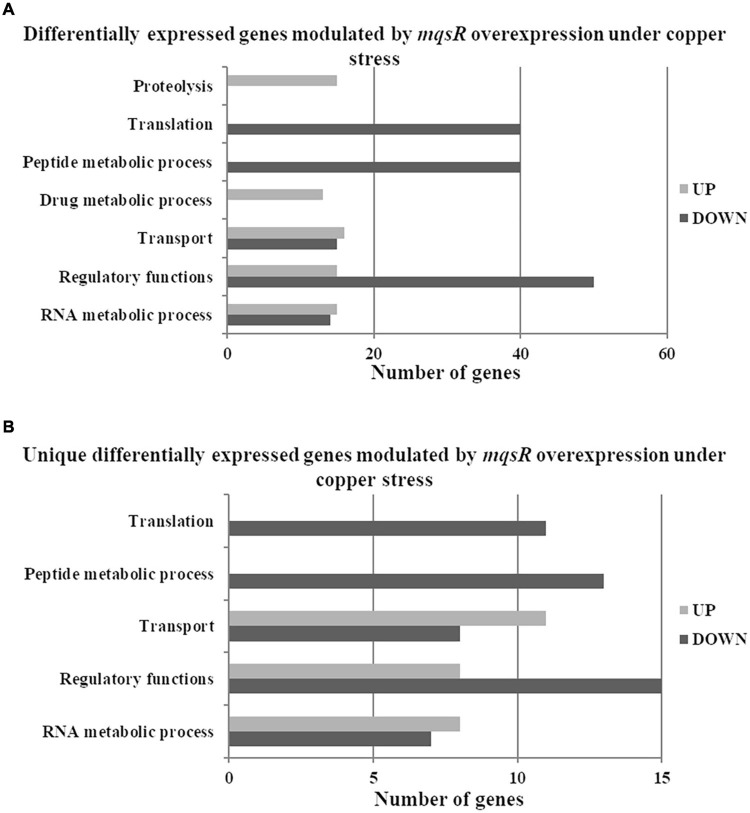
Gene Ontology categorization of differentially expressed genes in *X. fastidiosa* overexpressing *mqsR* in response to copper. **(A)** Genes were classified by functional category of biological processes using Blast2GO. **(B)** Categorization of differentially expressed genes unique to *Xf-mqsR* in response to copper. UP: upregulated genes; DOWN: downregulated genes.

Next, the functional characterization of the M-3/M-0 data set was performed to identify which genes are differentially modulated by the overexpression of MqsR under copper stress. RNA-Seq log_2_ fold-change values were confirmed by RT-qPCR for 10 selected genes selected from Table 2 with a Pearson correlation coefficient of 0.93 (Supplementary Figure 4). Genes related to translation and peptide metabolic processes were exclusively repressed in the M-3/M-0 libraries, whereas proteolysis and drug metabolic processes were induced (Figure 5A and Supplementary Material 1, Data Sets 8, 9). Other categories identified in this analysis included transport, regulatory functions, and RNA metabolic processes. Therefore, besides lowering the metabolism, the cells activate these specific salvage mechanisms allowing copper tolerance.

**TABLE 2:** Genes modulated by MqsR in *X. fastidiosa* under copper stress.

**Functional group**	**Gene name** *	**Locus Tag** **	**Protein**	**Product**	**LogFC**
**Peptide metabolic process**	Chaperone protein ClpB	XF_RS01600	WP_010892912.1	Chaperone protein ClpB	0.8
	Molecular chaperone GroES	XF_RS02575	WP_004088683.1	Molecular chaperone GroES	1.12
	Molecular chaperone DnaK	XF_RS10150	WP_010894786.1	Molecular chaperone DnaK	1.19
**Proteolysis**	Protease modulator HflC	XF_RS01875	WP_010892981.1	Protease modulator HflC	–0.946
	ATP-dependent protease	XF_RS05000	WP_010893691.1	ATP-dependent protease	–0.926
	ATP-dependent Clp protease proteolytic subunit	XF_RS05040	WP_010893698.1	ATP-dependent Clp protease proteolytic subunit	–0.197
	Peptidase S14	XF_RS02140	WP_042462775.1	Clp protease ClpP	1.70
**Toxins**	Hypothetical protein (Colicin V)	XF_RS01135	WP_010892803.1	Hypothetical protein	–1.40
**Regulatory functions**	Fis family transcriptional regulator	XF_RS13495	WP_010894455.1	Fis family transcriptional regulator	–1.55
	RNA polymerase-binding protein DksA	XF_RS04240	WP_010893509.1	RNA polymerase-binding protein DksA	0.93
	DNA-directed RNA polymerase subunit omega (*rpoZ*)	XF_RS06345	WP_010894003.1	DNA-directed RNA polymerase subunit omega	0.914
	HP	XF_RS07050	WP_042463203.1	HP (Helix-turn-helix XRE-family like proteins)	1.11
**Attachment/motility**					
**Afimbrial adhesins**	Surface protein (*hsf*)	XF_RS06465	WP_010894030.1	Surface protein	2.00
	Hemagglutinin (*pspA*)	XF_RS13660	WP_010894644.1	Filamentous hemagglutinin	2.21
**Fimbrial adhesin**	Fimbrial protein (*pilO*)	XF_RS01560	WP_010892902.1	Fimbrial protein	–1.87
**TA systems**	Addiction module antitoxin RelB	XF_RS07275	WP_042463224.1	Type II toxin-antitoxin system RelE/ParE family toxin	1.40
	Antitoxin (*mqsA*)	XF_RS10795	WP_010894926.1	Antitoxin	2.39
	HP (*mqsR*)	XF_RS10790	WP_010894925.1	Type II toxin-antitoxin system MqsR family toxin	0.83
**Transporters**	MFS transporter	XF_RS07585	WP_010894236.1	MFS transporter	1.49
	ion transporter	XF_RS06010	WP_010893927.1	Ion transporter	1.32
**Copper homeostasis**	Copper homeostasis protein CutC	XF_RS05650	WP_042463096.1	Copper homeostasis protein CutC	2.22

*Nomenclature according to GenBank.

**Locus Tag corresponds to GenBank accession numbers.

The proteolysis category included another peptidase S4 *clpP* (XF_RS02140) and *tldD* (XF_RS04775) metalloprotease, important regulators of bacterial metabolism. These genes are related to protein degradation. Although yet unclear, *tldD* was described as a putative regulator of chromosome-encoded TA system activities (Hu et al., 2012). Among the upregulated genes involved in transport, there were genes that encode ion transporters and sulfate transporters belonging to the ABC transporter family. ABC transporters are known to be involved in the influx or efflux of a wide diversity of molecules, and also with antimicrobial peptide resistance (Orelle et al., 2019). The categories associated with translation and peptide metabolic process showed downregulated genes encoding ribosomal subunits and the elongation factors EF-Tu and EF-G. Interestingly EF-Tu is described as the most enriched protein in *X. fastidiosa* outer membrane vesicles (OMVs) important for pathogen systemic dissemination throughout the host xylem vessels (Feitosa-Junior et al., 2019). The category linked to regulatory functions showed various downregulated genes, such as the global regulator *fis*, which is involved in virulence and pathogenicity. The rice pathogen *Dickeya zeae* showed remarkably decreased virulence capacity after *fis* deletion (Lv et al., 2018). This global virulence regulator is involved in exopolysaccharide production, motility, biofilm formation, and cellular aggregation in *Dickeya zeae*. All these processes are of utmost importance for *X. fastidiosa* pathogenicity, being associated with host colonization. The *rpoA* and a DNA-binding regulator hypothetical protein (XF_RS07050) were also repressed in the overexpressing strain under copper treatment.

To identify genes exclusively modulated by MqsR under copper stress, we analyzed the gene ontology of the 111 upregulated genes and 84 downregulated genes presented in Figures 4, 5B (Supplementary Material 1, Data Sets 4, 10, 11). The exclusively downregulated categories included translation and the peptide metabolic process (Figure 5B and Supplementary Material 1, Data Sets 10, 11). Other categories identified were transport, regulatory functions, and RNA metabolic process. The genes *dksA* and *rpoZ* from the regulatory functions group are transcriptional regulators associated with stress responses (Mathew and Chatterji, 2006; Wang et al., 2018) and were induced under copper stress. The highlighted genes that are modulated by MqsR under copper stress are listed in Table 2.

**FIGURE 6:**
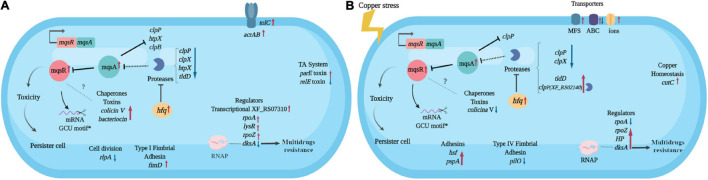
Hypothetical model of the *X. fastidiosa* MqsR-dependent regulon. Response to overexpression of *mqsR* in *X. fastidiosa* **(A)** and under copper stress **(B)**. Arrows pointing to the side (→) indicate direct regulation and up (↑) red arrows indicate upregulation, while ⊥ indicates inhibition and down (↓) blue arrows indicate downregulation. Dashes indicate direct binding and dotted lines indicate hypothetical regulations. *Demonstrated by Lee et al. (2014). The figure was created in BioRender.com.

Considering all the above-mentioned results, we built a hypothetical model for the *mqsR* overexpression and its influence on the *X. fastidiosa* regulatory mechanisms under normal and copper-induced stress conditions (Figure 6).

## Discussion

Copper-containing compounds are among the most used chemicals in agriculture (Lamichhane et al., 2018). The antimicrobial effects of copper were previously attributed to stress-induced responses in many bacterial plant pathogens (Wright et al., 2019; Martins et al., 2021), including *X. fastidiosa* (Rodrigues et al., 2008; Muranaka et al., 2012; Merfa et al., 2016; Ge et al., 2021). In this bacterium, the MqsRA TA system was reported to play a key role when the pathogen is under copper stress. MqsRA is likely to function as an indicator for exogenous stressors through the induction of cell elongation, formation of structured biofilm aggregations, and reduction in cell movement (Merfa et al., 2016). To better understand the roles the toxin MqsR may be playing over stress-induced responses in *X. fastidiosa*, we assessed the major phenotypic outcomes and the global transcriptional profile of the *mqsR*-overexpressing strain under copper-stress conditions through microscopy and RNA-Seq analysis.

The *mqsR* overexpression triggers genetic response where cells activate genes associated to stress adaptation (Figure 6A), from which many are conserved in the presence of copper (Figure 6B). These characteristics suggest that increasing the amount of MqsR leads to a priming effect of cells to stresses that normally induce expression of *mqsR*, like copper. We observed an approximately 10% increase in cell survival after copper treatment in the population overexpressing *mqsR* and considering that, in stationary phase, only up to ∼1% of cells are persisters (Keren et al., 2004; Lewis, 2007), we can infer that a higher number of persisters were present under this condition. Therefore, our results demonstrate that the presence of the stressor is not needed to induce the genes and consequent cell morphology changes when *mqsR* is overexpressed. These morphologies include the elongated cell formation and population heterogeneity, indicative of persister cell activation (Michiels et al., 2016; Fisher et al., 2017), which is supported by *tolC* induction (Figure 6A), that was associated with *E. coli* persistence (Pu et al., 2016). Indeed, the presented results fit perfectly in the mathematical model in which systems that do not present bistability produce the hysteretic switch to the persistent state (Fasani and Savageau, 2013), represented in our condition by the overexpression of *mqsR*.

Besides, the overexpression of *mqsR* in *E. coli* exhibited cellular toxicity, resulting in increased persister cell formation (Kim et al., 2010). Taken together and with previous results (Muranaka et al., 2012; Merfa et al., 2016), the role of mqsR in *X. fastidiosa* seems to be similar to *E. coli* which involves the induction of persister cells.

It has been demonstrated that the MqsRA TA system in *X. fastidiosa* likely autoregulates its own expression to balance the toxin and antitoxin in the most beneficial ratio for the cells to oppose the stress (Merfa et al., 2016). The *mqsR* overexpression itself presents a stress condition to the cell, thus to inactivate the toxin, the antitoxin MqsA should be produced to reach a T:A balance (Brown et al., 2013). Indeed, we observed an induction of *mqsA* under both conditions (Figures 6A,B). An upregulation of *hfq* in *Xf-mqsR* was observed in both conditions with and without copper stress. The *hfq* gene encodes an RNA chaperone that, among other regulatory functions, is related to the downregulation of proteases (Kim and Wood, 2010). It suggests that *hfq* is a key gene in the autoregulation of the MqsRA TA system, and we propose it could be one of the factors responsible for keeping the ideal T:A ratio in the cell by controlling the expression of proteases and consequently the cell morphologies observed in this work.

The genes modulated *clpP*, *hfq*, and *clpB* by MqsR in *X. fastidiosa* resemble those modulated by the same regulon in *E. coli* (Kim et al., 2010). These genes are involved in stress responses and contribute to toxicity and, consequently, to persister cell formation in *E. coli* (Kim and Wood, 2010; Kim et al., 2010). Differences in the global transcriptional profile were also observed, suggesting a potential *X. fastidiosa*-exclusive mechanism. Among the exclusive genes modulated by MqsR only in *X. fastidiosa* are two gene regulators (XF_RS07050 and XF_RS07310). The regulator XF_RS07310 has the same type of HTH domain as the MqsA antitoxin, suggesting that it could also bind to promoter regions of target genes and modulate their expression. Some regulators related to bacterial survival and stress responses previously described in several bacteria were also modulated (Maddocks and Oyston, 2008; Santiago et al., 2015; Merfa et al., 2016; Weiss et al., 2017).

The MqsA antitoxin regulates the expression of *mqsRA* and other genes in *E. coli* by binding to palindromic sequences in their promoter regions and repressing their expression (Brown et al., 2009; Wang and Wood, 2011; Soo and Wood, 2013). The MqsA antitoxin encoded by *X. fastidiosa* has the same amino acid residues in its HTH domain responsible for DNA binding (Merfa et al., 2016). Therefore, we searched for the MqsA-like palindromic sequence 5’-AAC (N)7 GTT in the genome of *X. fastidiosa* (Supplementary Table 3), seeking to identify those genes that were specifically differentially expressed in our RNA-Seq analyses. We investigated gene regulations in conditions where *mqsRA* expression is increased, such as under copper stress and *mqsR* overexpression. We verified 526 palindromic regions throughout the *X. fastidiosa* genome, with 77 corresponding to intergenic regions (Supplementary Table 3). Among the DEGs, a few showed the searched palindromic sequence in their intergenic regions (Supplementary Table 4). These genes included *clpP*, *htpX*, *clpB*, and *rpfB*, besides *mqsR* itself. According to data RNA-Seq, *clpP* and *clpB* genes remained downregulated, while *mqsA* expression remained upregulated, suggesting that MqsA may be regulating proteolysis under stress conditions in *mqsR* overexpression.

In our model, copper stress induces responses independent of MqsR involving protein degradation and multidrug resistance. When *mqsR* is overexpressed under copper stress, other *clpP* (XF_RS02140) and *tldD* encoding proteases were induced (Figure 6B). Thus, the observed upregulation of proteases could contribute to the consequent upregulation of *mqsRA*. The regulator *dksA*, which plays an important role in the multidrug resistance in *E. coli* (Wang et al., 2018), shifted from downregulation in normal growth conditions to upregulation under copper stress, supporting its role of multidrug resistance. Copper also induces the expression of transporter genes associated with multidrug efflux pumps including *cutC*, which is specific for copper efflux (Rodrigues et al., 2008; Li et al., 2009). It has been shown that multidrug efflux pumps induce persistence, and persister cells combine active efflux with passive numbness to survive antibiotic attacks (Pu et al., 2016). This demonstrates the interplay between resistance and tolerance mechanisms, which are complementary and redundant bacterial strategies to survive under stress conditions (Lewis, 2007).

Overall, with the results herein presented, we were able to expand the knowledge on the genes and mechanisms associated with MqsR, as well as the function of the MqsRA TA system in *X. fastidiosa*. MqsR regulates genes that alter cell behavior in order to prime them to respond to environmental stress, which is related to induction of persistence. The persistence in plant-pathogenic bacteria is an important tolerance mechanism to this agrochemical which is still neglected in the management of agricultural diseases.

## Data Availability Statement

The datasets generated and analyzed for this study can be found under the NCBI Bio-Project ID PRJNA718853. Other data used in this study are available on request from the corresponding author.

## Author Contributions

AS and MT conceived and designed this research, provided reagents, analytical tools, and revised the manuscript. IC and PM conducted the experiments and analyzed the data. IC, MM, NT-S, MT, and AS wrote the manuscript. IC, MM, PM, MT, and AS contributed to the interpretation of the data and provided intellectual input. All authors read and approved the final manuscript.

## Conflict of Interest

The authors declare that the research was conducted in the absence of any commercial or financial relationships that could be construed as a potential conflict of interest.

## Publisher’s Note

All claims expressed in this article are solely those of the authors and do not necessarily represent those of their affiliated organizations, or those of the publisher, the editors and the reviewers. Any product that may be evaluated in this article, or claim that may be made by its manufacturer, is not guaranteed or endorsed by the publisher.

## Supplementary Material

Supplementary Figure 1 *X. fastidiosa* transformed with the pXF20 empty vector.

Supplementary Figure 2 Experimental design.

Supplementary Figure 3 Reads of RNA-Seq.

Supplementary Figure 4 Validation RNA-Seq data.

Supplementary Table 1 Bacterial strains and plasmids.

Supplementary Table 2 Primers used for real-time quantitative PCR.

Supplementary Table 3 MqsA-like palindromic sequences (5’-AAC (N)7 GTT-3’) found in the genome of *X. fastidiosa* 9a5c.

Supplementary Table 4 Genes with the MqsA-like palindromic sequence identified in the RNA-Seq.

Supplementary Data Set 1 Differential gene expression analysis between the M-0/C-0.

Supplementary Data Set 2 Differential gene expression analysis between the M-3/M-0.

Supplementary Data Set 3 Differential gene expression analysis between the C-3/C-0.

Supplementary Data Set 4 Differential gene expression analysis between the downregulated and upregulated genes from the C-3/C-0 and M-3/M-0.

Supplementary Data Set 5 Venn diagram of differentially expressed genes in M-3/M-0 and C-3/C-0.

Supplementary Data Set 6 Functional characterization of proteins encoded by genes downregulated by MqsR in *X. fastidiosa* from Figure 3.

Supplementary Data Set 7 Functional characterization of proteins encoded by genes upregulated by MqsR in *X. fastidiosa* from Figure 3.

Supplementary Data Set 8 Functional characterization of proteins encoded by genes upregulated by MqsR in *X. fastidiosa* under copper stress from Figure 5A.

Supplementary Data Set 9 Functional characterization of proteins encoded by genes downregulated by MqsR in *X. fastidiosa* under copper stress from Figure 5A.

Supplementary Data Set 10 Functional characterization of proteins encoded by unique genes downregulated by MqsR in *X. fastidiosa* under copper stress from Figure 5B.

Supplementary Data Set 11 Functional characterization of proteins encoded by unique genes upregulated by MqsR in *X. fastidiosa* under copper stress from Figure 5B.
