# Supplementary material for: Overexpression of mqsR in Xylella fastidiosa Leads to a Priming Effect of Cells to Copper Stress Tolerance
Source: Front Microbiol. 2021 Sep 20;12:712564. doi: 10.3389/fmicb.2021.712564 (PMC8488296; doi:10.3389/fmicb.2021.712564)
Supplement: Supplementary Figure 1 — X. fastidiosa transformed with the pXF20 empty vector. [file Data_Sheet_2.docx]

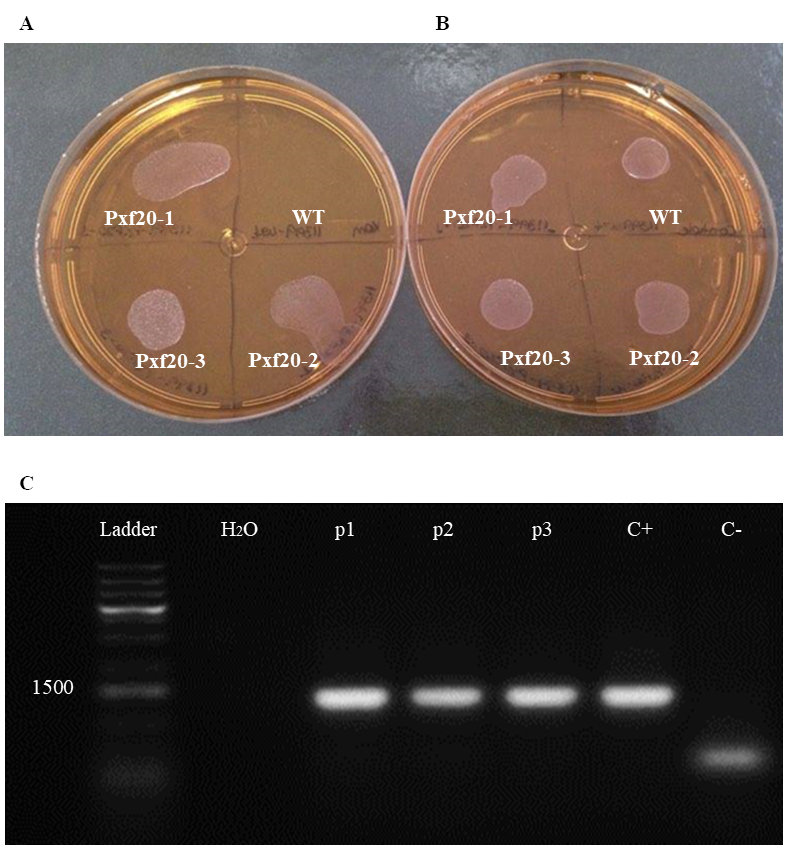


Figure S1. Selection and confirmation of *X. fastidiosa* transformed with the pXF20 empty vector. The obtained transformants were plated in PW containing kanamycin**(A)**, and in PW without this antibiotic (control) **(B)**. WT: wild-type (WT)*X. fastidiosa* strain 11399, used as negative control; pXF20-1 to -3: clones 1, 2 and 3 of the bacterium transformed with the pXF20 empty vector. **(C)**1% agarose gel of the PCR carried out with specific pairs of primers to detect the pXF20 empty vector in*X. fastidiosa* clones to confirm the transformation. The oriV and trfA ORFs of the pXF20 vector were amplified as a single amplicon. Ladder:GeneRuler 1 kb Plus DNA Ladder (Thermo Fisher Scientific, Waltham, Massachusetts, United States); H_2_O: non-template control PCR (negative control); p1, p2 and p3: PCRs carried out with the clones pXF20-1, pXF20-2 and pXF20-3, respectively; C+: PCR carried out with purified pXF20 vector (positive control); C-: PCR carried out with the WT strain 11399, which does not contain the pXF20 vector (negative control); Amplicon size: 1194 bp. Amplification of the specific amplicon only in the positive control and tested clones confirmed the transformation of *X. fastidiosa* strain 11399 with the pXF20 empty vector.


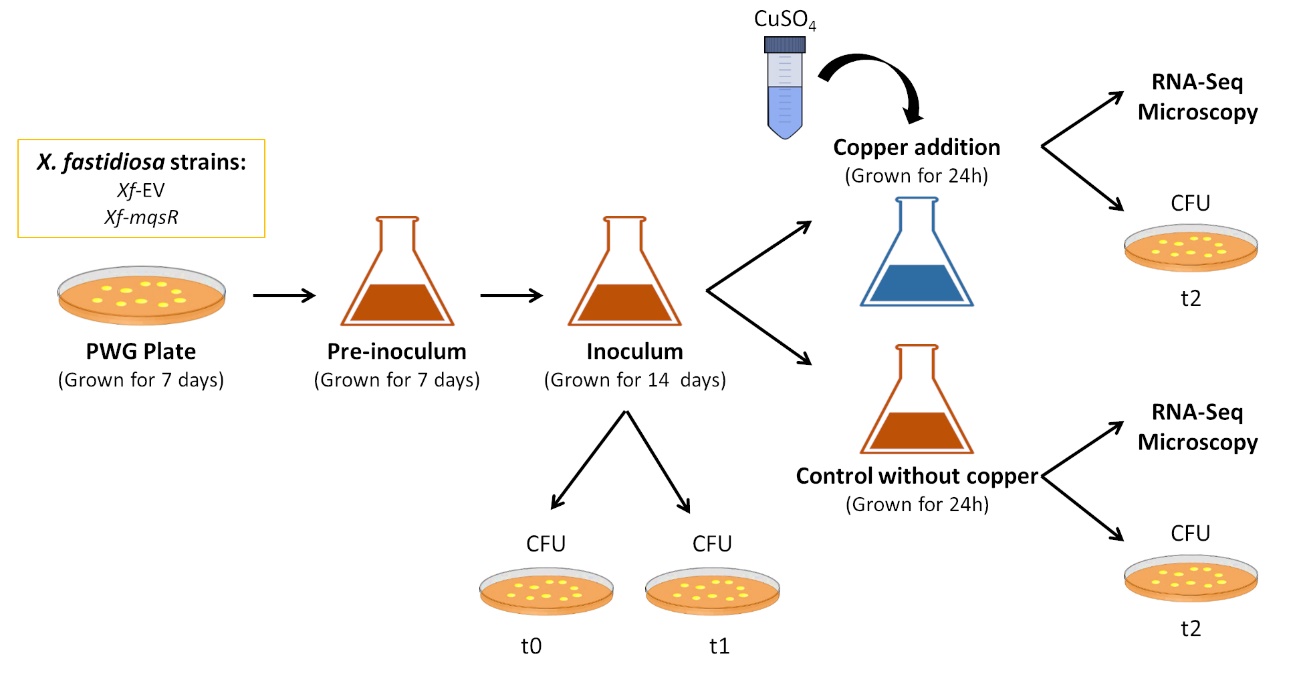


Figure S2. Experimental conditionsfor*X. fastidiosa*strains under copper-induced stress. All bacteria were grown in PWG plate for 7 days. After, to prepare the pre-inoculum,cells were collected and grown in PW broth for 7 days. The inoculum was prepared by adding the pre-inoculum into 90 mL of PW to an initial OD_600_ of 0.1 (t0) and incubating under shaking for 14 days (t1). Samples were treated with 3 mM CuSO_4_ and allowed to grow for additional 24 hours under copper stress (t2). The non-copper control samples of both strains were grown in pure PW broth. Aliquots of the entire experimental condition described above were collected to determine the CFU.mL^-1^ of each biological experiment at the following time course: inoculation time (t0), 14 days after growth in fresh PW broth when CuSO_4_ was added (t1) and 24 hours after copper sulfate treatment (t2), completing 15 days of growthl. From each sample a 10-fold serial dilution was performed and plated in PWG to estimate CFU. Four replicates were used for each sample, which were grown at 28 °C for 30 days.


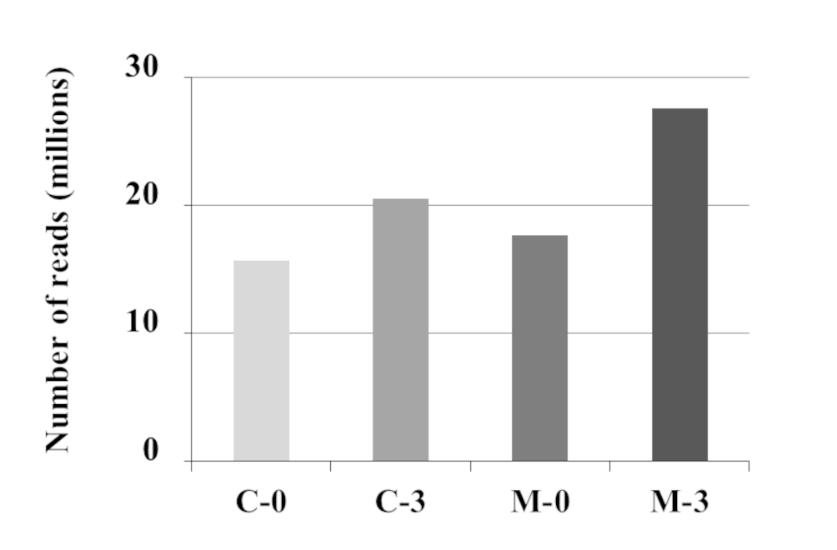


Figure S3. Reads of RNA-Seq. C-0: *Xf*-EV cells without copper treatment; C-3*: Xf*-EV cells treated with 3 mM CuSO4. M-0: *Xf-mqsR* cells without copper treatment. M-3: *Xf-mqsR* cells treated with 3mM CuSO4.This graphic represents average of each treatment in triplicate.


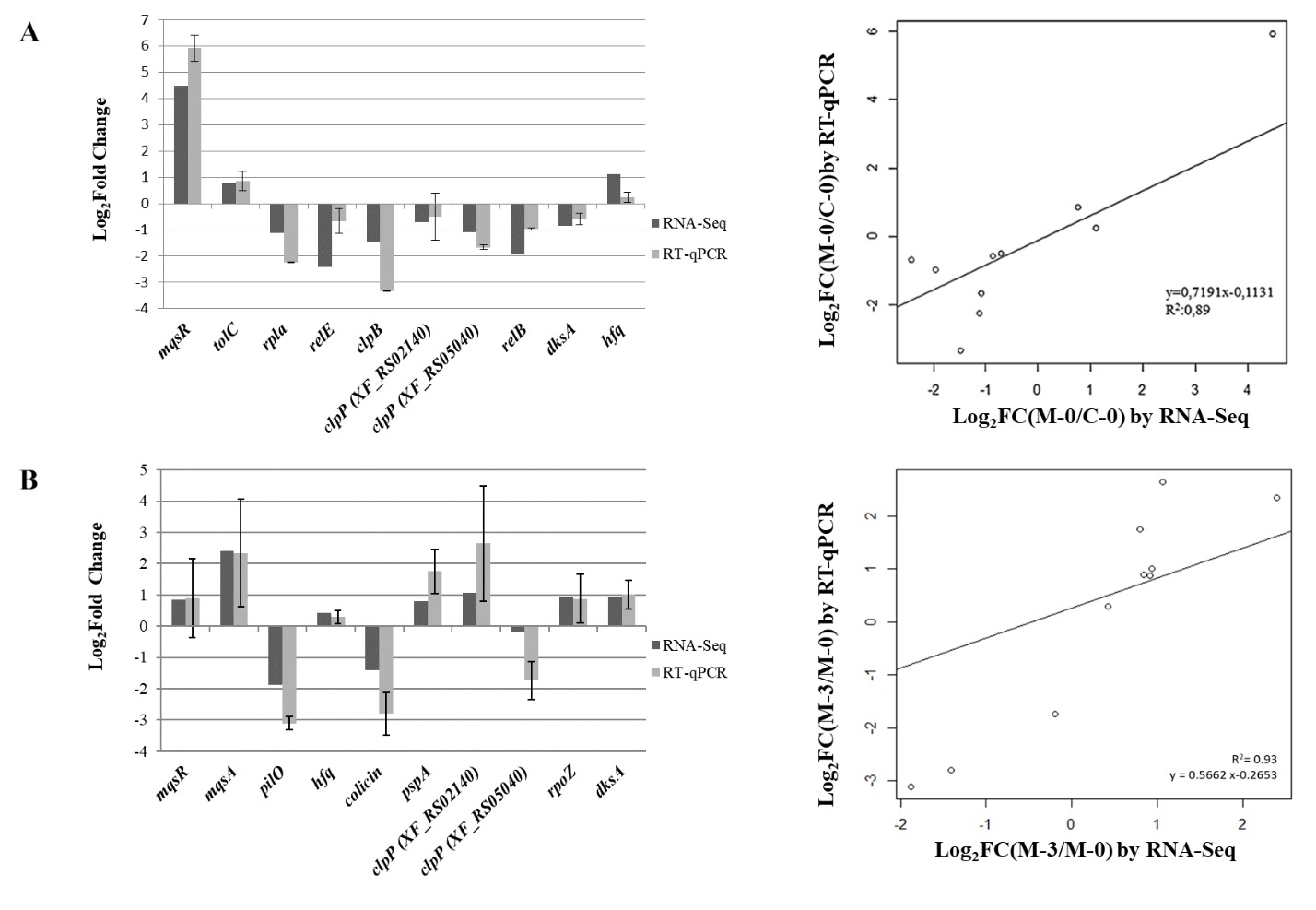


Figure S4. Validation RNA-Seq data. **(A)** RNA-Seq validation using RT-qPCR of the pairwise comparison of the M-0/C-0 libraries and correlation of the RT-qPCR and RNA-Seq data of the M-0/C-0 libraries. (**B**) RNA-Seq validation using RT-qPCR of the pairwise comparison of the M-3/M-0 libraries and correlation of the RT-qPCR and RNA-Seq data of the M-3/M-0 libraries. Log_2_ Fold Change of RT-qPCR data was plotted against Log_2_ Fold Change of RNA-Seq data for 10 genes. The equation of the line and the Pearson correlation coefficient (R^2^) are shown. Bars indicate standard errors. Mean of 3 biological replicates for experiments with RT-qPCR and RNA-Seq. Log_2_ Fold Change (Log_2_FC) values lower than 0 indicate downregulation of genes, whereas values greater than 0 indicate upregulation of genes.
